# Supplementary material for: Dynamic transcriptomic profiles of zebrafish gills in response to zinc supplementation
Source: BMC Genomics. 2010 Oct 11;11:553. doi: 10.1186/1471-2164-11-553 (PMC3091702; doi:10.1186/1471-2164-11-553)
Supplement: Additional file 2 — Interactive Direct Interaction Network representing the molecular interactions between zinc, copper, iron, calcium and proteins encoded by transcripts changed by zinc supplementation. Mini web-site containing index.html and hyperlinked pages in subdirectory describing a Direct Interaction Network automatically generated based on curated interactions contained within the proprietary PathwayArchitect database. Ovals represent proteins and the circles symbolize metal ions. Objects are coloured by their abundance in zebrafish at the time-point they were significantly different from the control is a scale from -4 fold (dark green) to +4 fold (dark red). Where significant differences were found at more than one time-point, the colour overlay shows expression at the first instance. Dark blue squares denote 'binding', and light blue squares 'expression'; green squares stand for 'regulation', green diamonds for 'metabolism', and green circles for 'promoter binding'. Arrow heads indicate directionality of the interaction where annotated. All nodes and edges can be further interrogated by selecting the relative area of the image. [file 1471-2164-11-553-S2.zip › PathwayArchitect Zn xs DIN/639578.html]

# REGULATION:

|  |  |
| --- | --- |
| Type | REGULATION |
| Effect | Positive |


---

|  |  |
| --- | --- |
| Score | 0 |


---

|  |  |
| --- | --- |
| Reference Count | 13 |


---

|  |  |
| --- | --- |
| Mechanism | Unknown |


---

|  |  |
| --- | --- |
| Reference:0 || Sentence | "Tetrodotoxin also had no effect on spontaneous or CRH-induced cytosolic Ca2+ levels." |
| PMID | 8641175 |
| Year | 1996 |
| Species | Rat |
| Journal | Endocrinology |
| RefScore | 1 |
| Source | PArchNLP |
  |
|  |  |


---

|  |  |
| --- | --- |
 Reference:1 || Sentence | "The L-type Ca2+ channel inhibitor nifedipine abolished spontaneous and CRH-induced action potentials and cytosolic Ca2+ transients, but did not eliminate the CRH-induced membrane depolarization or completely restore cytosolic Ca2+ to basal levels." |
| PMID | 8641175 |
| Year | 1996 |
| Species | Rat |
| Journal | Endocrinology |
| RefScore | 1 |
| Source | PArchNLP |
  ||  |  |


---

|  |  |
| --- | --- |
 Reference:2 || Sentence | "Inhibition of P-type Ca2+ channels with omega-agatoxin-IVA decreased action potential firing frequency and reduced the CRH-induced increase in cytosolic Ca2+." |
| PMID | 8641175 |
| Year | 1996 |
| Species | Rat |
| Journal | Endocrinology |
| RefScore | 1 |
| Source | PArchNLP |
  ||  |  |


---

|  |  |
| --- | --- |
 Reference:3 || Sentence | "CRH-induced Ca2+ entry also occurs independently of action potentials and is due to P-type, and possibly L-type, Ca2+ channels activated by the CRH-induced membrane depolarization." |
| PMID | 8641175 |
| Year | 1996 |
| Species | Rat |
| Journal | Endocrinology |
| RefScore | 1 |
| Source | PArchNLP |
  ||  |  |


---

|  |  |
| --- | --- |
 Reference:4 || Sentence | "Corticotropin releasing factor induced Ca(2+) flux into the cytoplasm, while urocortin Ca(2+) flux into the nucleus with a remarkable oscillatory effect." |
| PMID | 12615968 |
| Year | 2003 |
| Species | Human |
| Journal | J Cell Sci |
| RefScore | 1 |
| Source | PArchNLP |
  ||  |  |


---

|  |  |
| --- | --- |
 Reference:5 || Sentence | "Corticotropin releasing factor (CRF) induces a rapid, within seconds, and dose-dependent increase in the intracellular Ca2+ in both human and hamster melanoma cells." |
| PMID | 9762905 |
| Year | 1998 |
| Species | Human |
| Journal | FEBS Lett |
| RefScore | 1 |
| Source | PArchNLP |
  ||  |  |


---

|  |  |
| --- | --- |
 Reference:6 || Sentence | "This dual modulatory action of CRH on inward rectifier and voltage-gated Ca2+ channels better describes the observed CRH-induced effects." |
| PMID | 10988025 |
| Year | 2000 |
| Journal | J Theor Biol |
| RefScore | 2 |
| Source | PArchNLP |
  ||  |  |


---

|  |  |
| --- | --- |
 Reference:7 || Sentence | "It is suggested that an increase in the slow inward Ca2+ current induced by CRF plays an important role in its positive inotropic effect and that its effect differs from that of cardiotonic steroids." |
| PMID | 2341022 |
| Year | 1990 |
| Journal | Gen Pharmacol |
| RefScore | 0 |
| Source | PArchNLP |
  ||  |  |


---

|  |  |
| --- | --- |
 Reference:8 || Sentence | "We, therefore, examined the effects of selective destruction of dentate gyrus granule cells, which send excitatory glutaminergic inputs to subfields CA4, CA3 and CA2, on CRH expression in the PVN." |
| PMID | 1320216 |
| Year | 1992 |
| Species | Rat |
| Journal | Neuroendocrinology |
| RefScore | 1 |
| Source | PArchNLP |
  ||  |  |


---

|  |  |
| --- | --- |
 Reference:9 || Sentence | "CRF (2 nmol/l) increased cytosolic Ca2+ concentration under 2.8 mmol/l glucose, dependent upon extracellular Ca2+." |
| PMID | 10480603 |
| Year | 1999 |
| Species | Rat |
| Journal | Diabetes |
| RefScore | 1 |
| Source | PArchNLP |
  ||  |  |


---

|  |  |
| --- | --- |
 Reference:10 || Sentence | "In the whole-cell patch-clamp recording, CRF enhanced Ca2+ currents through L-type Ca2+ channels in a dose-dependent manner similar to that for depolarization." |
| PMID | 10480603 |
| Year | 1999 |
| Species | Rat |
| Journal | Diabetes |
| RefScore | 1 |
| Source | PArchNLP |
  ||  |  |


---

|  |  |
| --- | --- |
 Reference:11 || Sentence | "These results suggest that in single beta-cells of rat islets, CRF, through its own receptor, potentiates Ca2+ influx through the L-type Ca2+ channel by activation of the cAMP/protein kinase A signaling pathway." |
| PMID | 10480603 |
| Year | 1999 |
| Species | Rat |
| Journal | Diabetes |
| RefScore | 2 |
| Source | PArchNLP |
  ||  |  |


---

|  |  |
| --- | --- |
 Reference:12 || Sentence | Our data were as follows: (a) CRH-induced rapid phosphorylation of conventional PKCalpha and PKCbeta, accompanied by parallel increase of their concentration within nucleus. (b) CRH suppressed the phosphorylation of novel PKCdelta and PKCtheta;, which remained in the cytosol. (c) CRH-induced transient phosphorylation of atypical PKClambda and had no effect on PKCmu. (d) The effect of CRH on each PKC isoenzyme was blocked by a CRHR1 antagonist. (e) Blockade of conventional PKC phosphorylation inhibited CRH-induced calcium ion mobilization from intracellular stores as well as the CRH-induced apoptosis and Fas ligand production. |
| PMID | 15649420 |
| Year | 2005 |
| Species | Rat |
| Journal | Biochem Biophys Res Commun |
| RefScore | 0 |
| Source | PArchNLP |
  |


---

|  |  |
| --- | --- |
